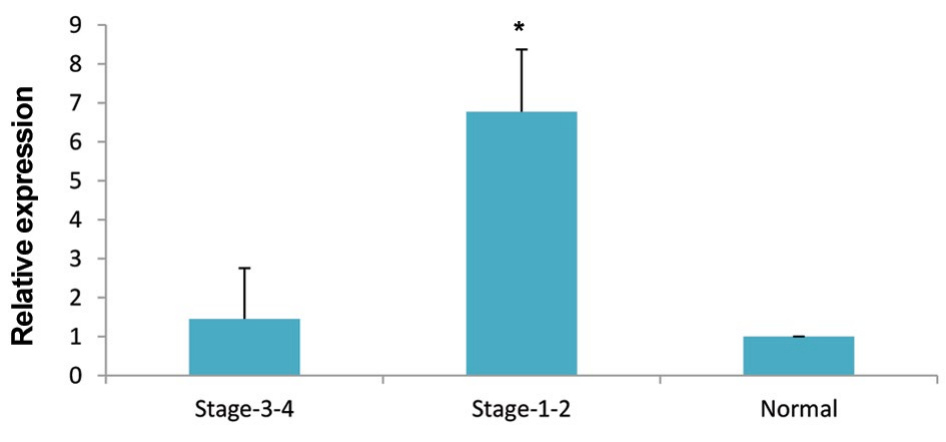# miR-31 and miR-145 as Potential Non-Invasive Regulatory Biomarkers in Patients with Endometriosis

**DOI:** 10.22074/cellj.2018.5850

**Published:** 2018-03-18

**Authors:** Oranous Bashti, Mehrdad Noruzinia, Masoud Garshasbi, Morteza Abtahi

**Affiliations:** 1 Department of Medical Genetics, Faculty of Medical Sciences, Tarbiat Modares University, Tehran, Iran; 2 Dena Hospital, Shiraz, Iran

This article published in Cell J _(Yakhteh)_, Vol 20, No 1, Apr-Jun 2018, on pages 84-89, the labels of columns in "Figure 3B" was changed. The correct one is presented below.

**Figure:**